# Effect of age and gender on ventricular-arterial coupling estimated using a non-invasive technique

**DOI:** 10.1186/s12871-024-02452-6

**Published:** 2024-02-27

**Authors:** Yurie Obata, Yuka Matsuki, Kazuhiro Okafuji, Kenji Shigemi

**Affiliations:** 1 Department of Anesthesiology, Yodogawa Christian Hospital, Osaka, Japan; 2 Department of Anesthesiology and Reanimatology, Faculty of Medicine Sciences, University of Fukui, Fukui, Japan; 3 Health Examination Center, Fukui-ken Saiseikai Hospital, Fukui, Japan; 4 Department of Anesthesiology and Reanimatology, Faculty of Medicine Sciences, University of Fukui, 23-3 Eiheijicho, Yoshidagun, 910-1193 Fukui Japan

**Keywords:** Ventricular-arterial coupling, End-systolic elastance, Arterial elastance, Non-invasive, Age, Gender

## Abstract

**Background:**

Left ventricular-arterial coupling is assessed as the ratio of left ventricular end-systolic elastance (Ees) to arterial elastance (Ea). Previous studies have introduced non-invasive estimations of Ees/Ea. It requires only four variables, namely pre-ejection period, ejection time, end-systolic pressure and diastolic pressure. The aims of the present study were to clarify the reference values of Ees/Ea estimated using the noninvasive technique, and to investigate the effects of age and gender on Ees/Ea in healthy subjects.

**Methods:**

This retrospective study utilized data from healthy, 30-79-year-old subjects. We recorded electrocardiogram, phonocardiogram, and brachial arterial pulse waves simultaneously using the vascular screening system, and used the observed variables to calculate Ees/Ea. We separated subjects into five groups according to their age and compared Ees/Ea among the different age groups.

**Results:**

The study included 2114 males and 2292 females. Ees/Ea ranged from 1.87 to 2.04 in males, and 1.98 to 2.32 in females. We observed no age-related differences in Ees/Ea in males (*p* = 0.10), and significant differences in females (*p* < 0.001). Ees/Ea in males was not different compared to those in females in 60-69-year-old group (*p* = 0.92). Whereas Ees/Ea was higher in females compared to those in males in the other age groups. The differences between medians of Ees/Ea in males and those in females were 0.45 (*p* < 0.001), 0.24 (*p* < 0.001), 0.13 (*p* = 0.01), and 0.13 (*p* = 0.03) in 30–39, 40–49, 50–59, and 70-79-year-old age groups, respectively.

**Conclusions:**

We clarified the reference values of Ees/Ea in healthy subjects. The effect of age on Ees/Ea is different in males and females, although Ees/Ea is maintained within a relatively narrow range in all subjects.

## Background

Left ventricular-arterial coupling (V-A coupling), which represents the interaction between the left ventricle and the arterial system, is an important factor determining cardiac performance. It is calculated as the ratio of measured left ventricular end-systolic elastance (Ees) to effective arterial elastance (Ea). Ees describes the slope and volume intercept of the left ventricular end-systolic pressure volume relationship [1]. Ees is indicative of left ventricular contractility, chamber geometry and passive ventricular stiffening [2]. Ea, a lumped index of arterial load, is calculated as end-systolic pressure divided by stroke volume [3, 4]. Ees and Ea have so far been assessed invasively, using a family of pressure-volume loops obtained during preload alteration [5]. However, as this entails invasive measurements, quantification takes time and appears impractical. Previous studies have described non-invasive estimations of Ees/Ea [6, 7] including a single beat method to estimate Ees/Ea using echocardiographic measurements [7]. While an ultrasound-based technique has a number of advantages, it has several limitations, e.g. difficulty in continuous real time monitoring and operator dependence, relying on the technical skills and the visual interpretation of the images. Hayashi et al. developed a technique to estimate Ees/Ea using four variables, namely arterial end-systolic pressure (Pes), arterial diastolic pressure (Pd), pre-ejection period (PEP) and ejection time (ET) [8]. These four variables can be obtained using electrocardiogram (ECG), phonocardiogram, pulse waveform, and non-invasive blood pressure. This method enables the measurement of Ees/Ea noninvasively, continuously and in real time, since it does not require measurement of left ventricular volume by echocardiography. However, Ees/Ea monitoring has still not been used in a clinical setting and its reference values are still unknown.

The aims of the present study were to determine the reference values of Ees/Ea estimated using the noninvasive technique, and to investigate the effects of age and gender on Ees/Ea in healthy subjects.

## Methods

This retrospective study utilized data from subjects who underwent vascular screening as a part of medical checkups at Fukui-ken Saiseikai Hospital between July 2007 and August 2017. The study protocol was approved by the Research Ethics Committee of Fukui-ken Saiseikai Hospital (No.2017-010). Written informed consent was obtained from all subjects. We enrolled males and females between the ages of 30 and 79 years. We excluded the following subjects: those with hypertension, diabetes mellitus, dyslipidemia, coronary heart disease, stroke, and the other cardiovascular diseases. Then we classified the subjects into five groups according to their age.

### Measurements

We recorded age, gender, height, and weight of the subjects. All subjects were placed in the supine position. A standard four lead ECG was attached, and a stethoscope was placed on the second right sternal border. Four blood pressure cuffs were placed around the arms and ankles. We simultaneously recorded the subjects’ ECG, phonocardiogram, brachial arterial pulse waves and tibial arterial pulse waves using the vascular screening system, Vasera VS-2000 (Fukuda Denshi Co. Ltd, Tokyo, Japan). Pre-ejection period (PEP), ejection time (ET), systolic blood pressure (SBP) and diastolic blood pressure (DBP) were automatically recorded by the device. ET was defined as the time difference between the upstroke and the dicrotic notch of the right brachial pulse wave. PEP was obtained by subtracting ET from the time between the Q wave and the second heart sound. SBP and DBP were determined by cuff sphygmomanometry. We calculated Pes from SBP and DBP using the formula published by Kappus et al. [9].

1 $${\text{Pes}} = \left(0.205\times{\text{SBP}}\right)\,+\,\left(0.898\times{\text{DBP}}\right)\,+\,0.4214.$$

Then, we calculated the ratio of Ees to Ea using the values of PEP, ET, Pes and DBP, as described previously [8]. Briefly, we solved the following simultaneous equations using Newton’s method and estimated Ees/Ea.

2 $${\text{Ees/Ea}} = {\text{Pd/Pes}} \left(1 + {\text{k}} \times {\text{ET/PEP}}\right) {-} 1.$$

3 $${\text{k}} = 0.53 \left({\text{Ees/Ea}}\right)^{0.51}.$$

In the above equations, k is the slope ratio of two straight lines in the ventricular time-varying elastance curve, one for the isovolumic phase and the other for the ejection phase. Equation (2) is algebraically expressed using the concept of the left ventricular pressure-volume relationship. Equation (3) is the empirical relationship between Ees/Ea and k, which was determined by extensively altering heart rate, contractility and afterload in dogs. We used DBP instead of Pd for the calculations.

### Statistical analysis

We report continuous variables as the median (interquartile range, IQR) and categorical variables as proportions. We identified and excluded the outliers of Ees/Ea using ROUT method [10]. Then the Kruskal-Wallis test and post hoc Dunn’s multiple comparison test were performed to compare Ees/Ea measured in each age group. Furthermore, the Mann-Whitney test was used to compare the demographic variables as well as Ees/Ea in males and females separately in each age group. Statistical significance was set at *p* < 0.05 and all tests were two sided.

## Results

**Fig. 1:**
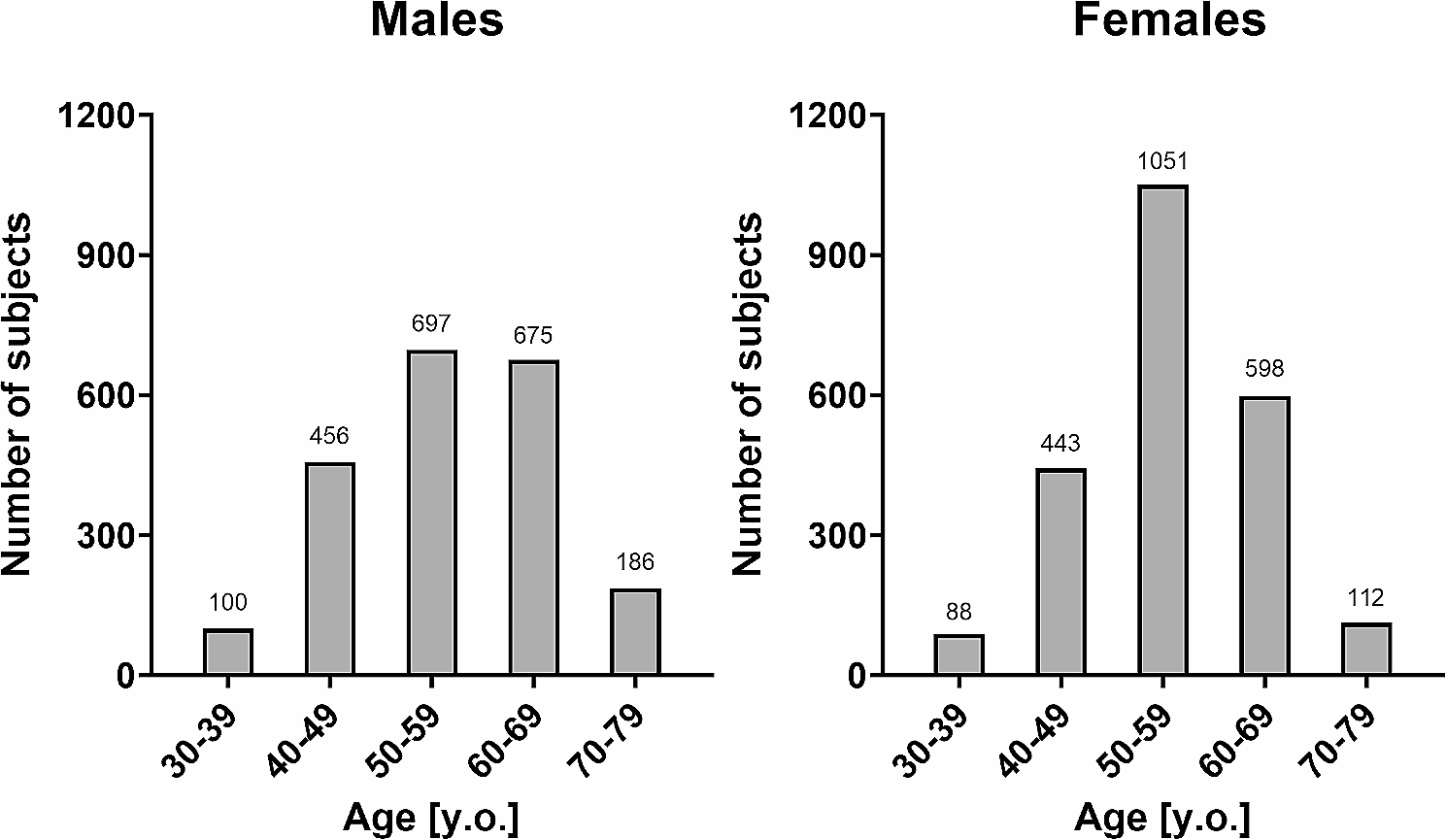
Number of males and females. Each bar indicates the number of males and females in each age group. The actual numbers are shown on the bars

A total of 8225 subjects were enrolled and 4406 subjects (2114 males and 2292 females) matched the including criteria. Figure 1 shows the number of subjects in each age group in males and females. The subjects’ demographics and baseline characteristics in each age group are presented in Table 1. Height and weight tended to decrease with age in each gender. The values of height, weight, and body mass index (BMI) were higher in males compared to females in all age groups. SBP and DBP tended to increase with age in each gender. SBP and DBP were higher in males compared to females except 70-79-year-old age groups.

**Table 1:** Demographic characteristics and blood pressure of the subjects in each age group

	Height [cm]			Weight [kg]			BMI [kg/m^2^]		
	Males	Females		Males	Females		Males	Females	
**Age [y.o.]**	Median (IQR)	Median (IQR)	**p value**	Median (IQR)	Median (IQR)	**p value**	Median (IQR)	Median (IQR)	**p value**
30–39	173 (169, 178)	159 (156, 161)	< 0.001	71 (65, 80)	51 (46, 58)	< 0.001	24 (22, 26)	20 (18, 22)	< 0.001
40–49	172 (168, 176)	159 (155, 162)	< 0.001	69 (63, 76)	54 (49, 61)	< 0.001	23 (22, 26)	21 (20, 24)	< 0.001
50–59	170 (167, 174)	158 (154, 161)	< 0.001	68 (62, 74)	53 (49, 59)	< 0.001	24 (22, 25)	22 (20, 24)	< 0.001
60–69	168 (164, 171)	155 (152, 158)	< 0.001	64 (59, 70)	51 (47, 56)	< 0.001	23 (21, 25)	21 (20, 23)	< 0.001
70–79	166 (161, 169)	153 (149, 156)	< 0.001	61 (55, 69)	47 (44, 53)	< 0.001	23 (21, 25)	21 (19, 23)	< 0.001
**All**	169 (166, 174)	157 (154, 160)	< 0.001	67 (61, 73)	52 (48, 58)	< 0.001	23 (22, 25)	21 (20, 23)	< 0.001
	**SBP [mmHg]**			**DBP [mmHg]**					
	**Males**	**Females**		**Males**	**Females**				
**Age [y.o.]**	Median (IQR)	Median (IQR)	**p value**	Median (IQR)	Median (IQR)	**p value**			
30–39	116 (111, 124)	104 (97, 112)	< 0.001	71 (66, 77)	63 (58, 69)	< 0.001			
40–49	117 (110, 124)	110 (104, 120)	< 0.001	75 (69, 81)	69 (62, 75)	< 0.001			
50–59	120 (111, 130)	114 (105, 123)	< 0.001	79 (72, 86)	71 (64, 78)	< 0.001			
60–69	120 (113, 130)	116 (107, 128)	< 0.001	78 (72, 85)	71 (65, 79)	< 0.001			
70–79	123 (115, 130)	121 (112, 133)	0.44	77 (71, 82)	76 (67, 80)	0.03			
**All**	119 (112, 128)	113 (105, 124)	< 0.001	77 (71, 84)	71 (64, 78)	< 0.001			

IQR: interquartile range; BMI: body mass index; SBP: systolic blood pressure; DBP: diastolic blood pressure

**Fig. 2:**
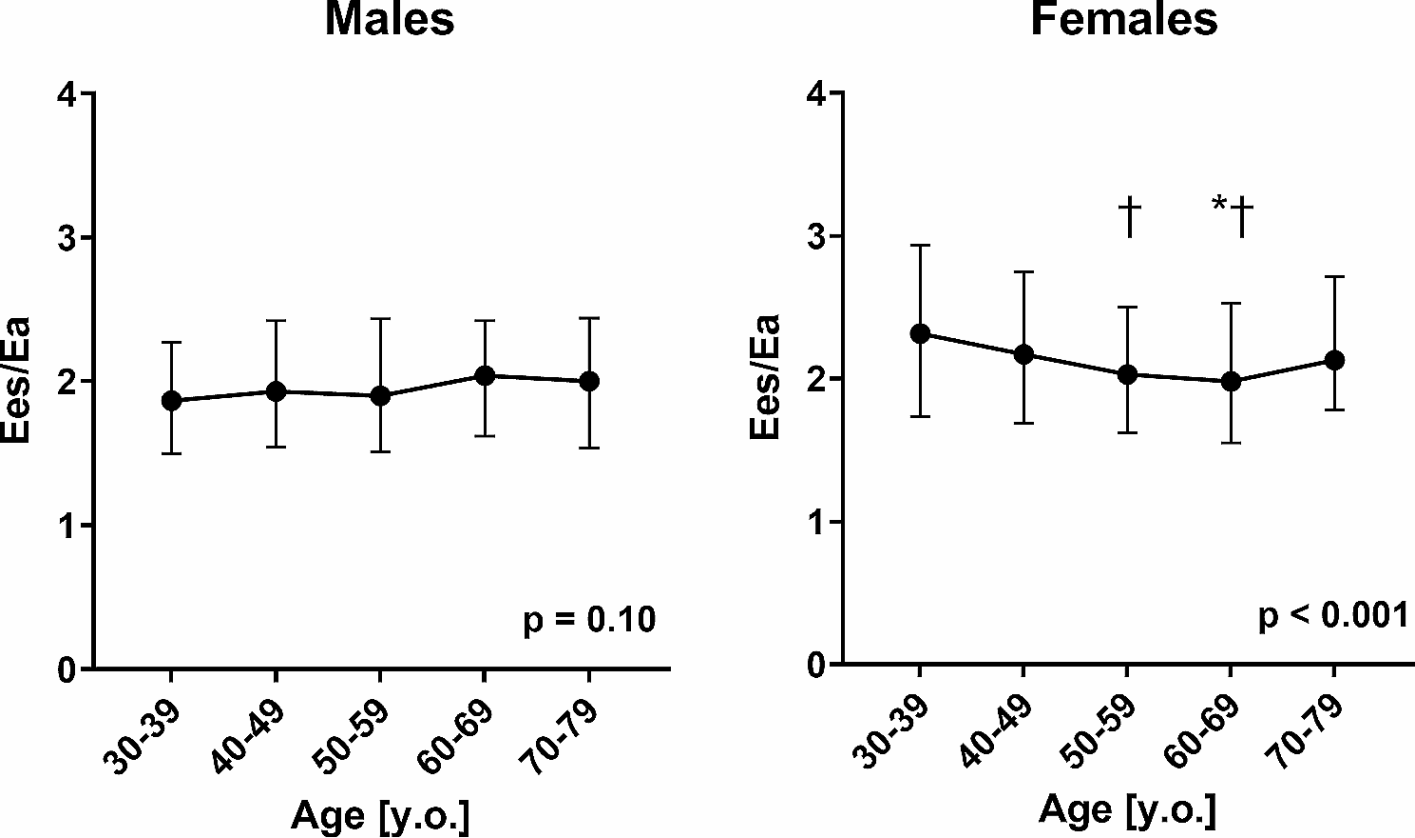
Effect of age on Ees/Ea. Changes in Ees/Ea with age in males and females. The points and whiskers indicate the median and interquartile range of Ees/Ea in each age group. The p value estimated by the Kruskal-Wallis test is presented in the lower right corner. ⋆ indicates a significant difference from the group of 30–39 year olds. ✝ indicates a significant difference from the group of 40–49 year olds

Median (IQR) values of Ees/Ea in each age group and for each gender are shown in Table 2. Ees/Ea ranged from 1.87 to 2.04 in males, and 1.98 to 2.32 in females. Figure 2 shows a comparison of Ees/Ea by age in males and females. We observed no differences in Ees/Ea in each group in males (*p* = 0.10). Whereas there were significant differences in Ees/Ea between females aged 30–39 years old and those aged 60–69 years old (*p* = 0.03), and between 40-49-year-old and 50–59 and 60-69-year-old females (*p* = 0.002 and *p* < 0.001 respectively). Ees/Ea was lowest in 60-69-year-old females. Table 2 also shows a comparison between Ees/Ea in males and females in each age group. We observed no gender related differences in Ees/Ea in 60-69-year-old age groups (*p* = 0.92). Ees/Ea was significantly higher in females compared to males in the 30–39, 40–49, 50–59, and 70-79-year-old age groups.

**Table 2:** Ees/Ea in each age group

	Males	Females	
**Age [y.o.]**	Median (IQR)	Median (IQR)	**p value**
30–39	1.87 (1.50, 2.27)	2.32 (1.73, 2.94)	< 0.001
40–49	1.93 (1.54, 2.42)	2.17 (1.69, 2.75)	< 0.001
50–59	1.90 (1.51, 2.44)	2.03 (1.62, 2.50)	0.01
60–69	2.04 (1.62, 2.42)	1.98 (1.55, 2.53)	0.92
70–79	2.00 (1.54, 2.44)	2.13(1.78, 2.72)	0.03
**All**	1.95 (1.54, 2.42)	2.05 (1.62, 2.58)	< 0.001

IQR: interquartile range

## Discussion

In the present study, we report the reference values of Ees/Ea in healthy subjects, estimated using a noninvasive method based on four variables, Pes, Pd, PEP and ET. We found that the effect of age on Ees/Ea is slightly different between males and females. Ees/Ea is lower in middle-aged females compared to those in young females, while Ees/Ea is constant regardless of age in males. However, the most important finding of this study is that Ees/Ea is maintained within a relatively narrow range over all age groups and in both genders.

As arterial stiffening and Ea increase with aging, the elastance of the left ventricle (Ees) increases proportionately to ensure adequate systolic V-A coupling and to optimize the transfer of blood from the heart to the arteries [2]. It has been shown that an age-related increase in Ea is associated with an increase in ventricular Ees measured using an invasive technique [11, 12]. As a result, the ratio of Ees to Ea is maintained unchanged irrespective of age. However, these previous studies did not focus on the effect of gender, and the majority of subjects in their study populations were males. As in previous studies, we found no difference in Ees/Ea in males.

Previous investigations on the effect of gender on V-A coupling have shown conflicting results. Najjar et al. assessed V-A coupling using gated radionuclide blood pool scans [13]. They showed that V-A coupling did not change regardless of age and gender. Redfield et al. measured V-A coupling using the modified single beat method in a large community-based population [14]. They showed that V-A coupling (Ea/Ees) did not change with age in males, but declined with age in females both in the entire study population and in those without cardiovascular disease. Coutinho et al. compared V-A coupling separately in males and females, using the same noninvasive method [2]. In their study, mean age was 65 years in males and 67 years in females. They showed that V-A coupling (Ea/Ees) was not different in males and females, although they included subjects with multiple cardiovascular risk factors. In our study, Ees/Ea remained unchanged with age in males, which is consistent with the results of previous studies. On the other hand, the change in Ees/Ea with age in females showed the opposite trend compared to the study by Redfield et al. However, they included a population older than 45 years and assessed changes in V-A coupling using a linear regression model. In our study, we included younger subjects and found that the changes in Ees/Ea followed a gradual U-shaped curve. We speculate that this age-related changes in Ees/Ea associate with the changes in the female hormonal environment. The acceleration of age-associated declines in vascular function in females after menopause has been reported in some studies [15, 16]. It may affect the increase in Ea and decrease in Ees/Ea. Also, we showed that SBP and DBP were lower in females compared to males in each age group. It could be one of the reasons why Ees/Ea in females were higher than those in males.

It should be noted that the median value of Ees/Ea in this study is slightly higher compared to those previously reported. Starling measured Ees/Ea in twenty-nine healthy patients using radionuclide angiography [5]. He showed that the normal human heart operates at an Ees/Ea ratio of 1.62 ± 0.80 under basal hemodynamic conditions. Hayward et al. assessed the left ventricular pressure volume loop using simultaneous conductance catheter volumetry, and measured Ees/Ea in 48-75-year-old subjects with normal left ventricular function [17]. They showed that the value of Ees/Ea, representing V-A coupling, was 1.19 ± 0.40 in females and 1.54 ± 0.30 in males. In our study the median value of Ees/Ea was around 2.0. One of the reasons why Ees/Ea in our study is higher than those in the previous studies is due to the method of the ET measurement. We measured ET using the vascular screening system which defined ET as the time difference between the upstroke and the dicrotic notch of the right brachial pulse wave. We have published previously that ET measured at the peripheral site is longer than the actual ET [18]. The longer ET makes the higher Ees/Ea. However, we believe that our results are still meaningful and reasonable. Burkhoff and Sagawa have shown that maximal myocardial efficiency, defined as the ratio of LV stroke work to myocardial oxygen consumption, occurs when the ratio of Ees to Ea is close to 2.0 [19].

The concept of V-A coupling was established around 30 years ago. Subsequently, the invasive experiments were performed around for 10 years. Half of previous studies we cited were published more than 15 years ago. Due to the requirement of the invasive measurements, V-A coupling is not used commonly in the operating room (OR) and in the intensive care unit (ICU) even though some noninvasive methods have been introduced. However, the concept of V-A coupling is important to understand the hemodynamics and to make an appropriate decision. For instance, when the vasopressors are used for maintaining arterial pressure in patients with septic shock, the effect can be detrimental by increasing the left ventricular workload and worsening V-A coupling [20]. In another example, a pre-existing condition of V-A decoupling because of a covert loss of myocardial contractility can be high risk of decompensation in case of pharmacological heart rate reduction [21]. Our study provided the reference values of V-A coupling using a noninvasive method. It could be the first step to use this method and assess V-A coupling in the OR and the ICU.

Our study has several limitations. The sample size in young and elderly age groups are relatively small. We focused on the healthy subjects and did not assess the effect of cardiovascular diseases on Ees/Ea. On the other hand, the elderly subjects may have undiagnosed or asymptomatic cardiovascular diseases. Further study is needed to clarify the prognostic value of Ees/Ea in patients with such diseases. We analyzed the data retrospectively without assessing the actual pulse waves and phonocardiogram. Arrhythmia, respiratory sounds, and motion artifacts might affect PEP/ET measurements. However, we included a large population and evaluated the median values of the measurements to minimize the effects of the measurement error. We did not perform transthoracic echocardiography in this cohort. Therefore, we cannot compare our results to the values of Ees/Ea estimated using the other noninvasive methods.

## Conclusions

In conclusion, we clarified the age-related reference values of Ees/Ea, calculated using formulas that included four noninvasively-determined variables. The effect of age on Ees/Ea is different in males and females, although Ees/Ea is maintained within a relatively narrow range in all subjects. Our findings will be useful in the clinical monitoring of Ees/Ea continuously and in real time.

## Data Availability

The data that support the findings of this study are available on request from the corresponding author.

## References

[1] Kass DA, Maughan WL (1988). From ’emax’ to pressure-volume relations: a broader view. Circulation.

[2] Coutinho T, Borlaug BA, Pellikka PA, Turner ST, Kullo IJ (2013). Sex differences in arterial stiffness and ventricular-arterial interactions. J Am Coll Cardiol.

[3] Chirinos JA (2013). Ventricular-arterial coupling: invasive and non-invasive assessment. Artery Res.

[4] Chemla D, Antony I, Lecarpentier Y, Nitenberg A (2015). Contribution of systemic vascular resistance and total arterial compliance to effective arterial elastance in humans. Am J Physiol Heart Circ Physiol.

[5] Starling MR (1993). Left ventricular-arterial coupling relations in the normal human heart. Am Heart J.

[6] Asanoi H, Sasayama S, Kameyama T (1989). Ventriculoarterial coupling in normal and failing heart in humans. Circ Res.

[7] Chen CH, Fetics B, Nevo E, Rochitte CE, Chiou KR, Ding PYA (2001). Noninvasive single-beat determination of left ventricular end-systolic elastance in humans. J Am Coll Cardiol.

[8] Hayashi K, Shigemi K, Sishido T, Sugimachi T, Sunagawa M (2000). Single-beat estimation of ventricular end-systolic elastance-effective arterial elastance as an index of ventricular mechanoenergetic performance. Anesthesiology.

[9] Kappus RM, Ranadive SM, Yan H, Lane AD, Cook MD, Hall G (2013). Validity of predicting left ventricular end systolic pressure changes following an acute bout of exercise. J Sci Med Sport.

[10] Motulsky HJ, Brown RE. Detecting outliers when fitting data with nonlinear regression - a new method based on robust nonlinear regression and the false discovery rate. BMC Bioinformatics. 2006; 7–123. 10.1186/1471-2105-7-123 PMC1472692 16526949

[11] Chen CH, Nakayama M, Nevo E, Fetics BJ, Maughan WL, Kass DA (1998). Coupled systolic-ventricular and vascular stiffening with age. J Am Coll Cardiol.

[12] Kawaguchi M, Hay I, Fetics B, Kass DA (2003). Combined ventricular systolic and arterial stiffening in patients with heart failure and preserved ejection fraction: implications for systolic and diastolic reserve limitations. Circulation.

[13] Najjar SS, Schulman SP, Gerstenblith G, Fleg JL, Kass DA, O’Connor F (2004). Age and gender affect ventricular-vascular coupling during aerobic exercise. J Am Coll Cardiol.

[14] Redfield MM, Jacobsen SJ, Borlaug BA, Rodeheffer RJ, Kass DA (2005). Age- and gender-related ventricular-vascular stiffening: a community-based study. Circulation.

[15] Moreau KL, Hildreth KL. Vascular aging across the menopause transition in healthy women. Adv Vasc Med. 2014; 2014:1–12. 10.1155/2014/204390 PMC4433172 25984561

[16] Pezel T, Michos ED, Varadarajan V, Shabani M, Venkatesh BA, Vaidya D (2022). Prognostic value of a left atrioventricular coupling index in pre- and post-menopausal women from the multi-ethnic study of atherosclerosis. Front Cardiovasc Med.

[17] Hayward CS, Kalnins WV, Kelly RP (2001). Gender-related differences in left ventricular chamber function. Cardiovasc Res.

[18] Obata Y, Mizogami M, Singh S, Nyhan D, Berkowitz DE, Steppan J, Barodka V (2017). Ejection time: influence of hemodynamics and site of measurement in the arterial tree. Hypertens Res.

[19] Burkhoff D, Sagawa K (1986). Ventricular efficiency predicted by an analytical model. Am J Physiol.

[20] Guarracino F, Bertini P, Pinsky MR (2019). Cardiovascular determinants of resuscitation from sepsis and septic shock. Crit Care.

[21] Morelli A, Romano SM, Sanfilippo F, Santonocito C, Frati G, Chiostri M (2020). Systolic-dicrotic notch pressure difference can identify tachycardic patients with septic shock at risk of cardiovascular decompensation following pharmacological heart rate reduction. Br J Anaesth.

